# BIX‐01294 enhanced chemotherapy effect in gastric cancer by inducing GSDME‐mediated pyroptosis

**DOI:** 10.1002/cbin.11395

**Published:** 2020-06-08

**Authors:** Bei‐Bei Deng, Bao‐Ping Jiao, Yang‐Jun Liu, Yan‐Rong Li, Gui‐Jun Wang

**Affiliations:** ^1^ Department of Clinical Laboratory The First Affiliated Hospital of Jinzhou Medical University Jinzhou China; ^2^ Department of General Surgery The First Affiliated Hospital of Jinzhou Medical University Jinzhou China; ^3^ Department of Gastroenterology The First Affiliated Hospital of Jinzhou Medical University Jinzhou China

**Keywords:** autophagy, gastric cancer, GSDME, pyroptosis

## Abstract

Adjuvant chemotherapy in combination with surgery is expected to be a curative strategy for gastric cancer. However, drug resistance remains an obstacle in effective chemotherapy. Therefore, understanding the potential mechanisms of chemotherapy induced gastric cancer cell death is of great importance. We demonstrated that BIX‐01294 (BIX) at low concentration could induce autophagic flux by converting LC3B‐I to LC3B‐II and directly activate autophagy associated cell death in gastric cancer cell lines at high concentration. BIX at low concentration could help obtain sensitivity of gastric cancer cells to chemotherapy with significantly reduced cell viability. Interestingly, BIX combined Cis (BIX + Cis) treated SGC‐7901 cells display pyroptosis related cell death with large bubbles blown around the membrane, significantly decreased cell viability, elevated lactate dehydrogenase release and increased percentage of propidium iodide and Annexin‐V double positive cells. Furthermore, the cleavage of gasdermin E (GSDME) and caspase‐3 but not GSDMD was detected by immunoblotting and the knockout of *GSDME* switched pyroptosis into apoptosis in the BIX + Cis combined treated group. Furthermore, the deficiency of *Beclin‐1* to inhibit BIX induced autophagic flux completely blocked BIX + Cis combined treated induced cell pyroptosis related cell death. Additionally, BIX + Cis in vivo treatment could inhibit tumor growth, which could be reversed by the deficiency of *Beclin‐1* and be delayed by the deficiency of *GSDME*. In conclusion, our data was the first to reveal that BIX enhanced the anticancer chemotherapy effect by induced GSDME‐mediated pyroptosis through the activation of autophagic flux in gastric cancer cells.

**Abbreviations**
BIX: BIX‐01294
BIX + Cis: BIX combined Cis
CCK‐8: Cell Counting Kit‐8 mixture
Cis: cis‐platinum
Cleaved CASP3: cleavage of caspase‐3
EHMT2: euchromatic histone‐lysine *N*‐methyltransferase 2
FBS: fetal bovine serum
GSDMD: gasdermin D
GSDMD‐F: full length of GSDMD
GSDMD‐N: N‐terminal of GSDMD
GSDME: gasdermin E
GSDME‐F: full length of GSDME
GSDME‐N: N‐terminal of GSDME
HMTase: EHMT2 histone methyltransferase
KO: knockout
LDH: lactate dehydrogenase
PFD: pore‐forming domain
PI: propidium iodide
RPMI: Roswell Park Memorial Institute
sgRNA: single guide RNA
WT: wild type

## 1 BACKGROUND

Gastric cancer accounts for the fifth most frequently diagnosed cancer (over 1,000,000 new cases) and is the third leading cause of cancer death (about 783,000 deaths) in the world, according to the Global Cancer Statistics 2018, and there will be about 18.1 million new cancer cases and 9.6 million cancer deaths in 2018 (Bray et al., 2018; Sulli et al., 2018). In China, the incidence rates of gastric cancer are markedly elevated and gastric cancer constitutes the second leading cause of cancer‐related death (Bray et al., 2018; Chen et al., 2016). Currently, adequate surgical resection is still expected to be the most curative option for gastric cancer, while chemotherapy or neoadjuvant therapy is usually applied in combination with surgery for locally advanced cancer to prevent recurrence and metastasis after surgery (Van Cutsem, Sagaert, Topal, Haustermans, & Prenen, 2016), it is broadly accepted that chemotherapy induces gastric cell death, and drug resistance and tumor growth are influenced by drug‐induced cancer cell death. Therefore, understanding the mechanism of chemotherapy‐induced gastric cancer cell death is eagerly awaited.

Autophagy, a conserved cellular self‐digestive process for maintaining homeostasis, degrades proteins and organelles through lysosomal degradation (Levy, Towers, & Thorburn, 2017). However, recent studies have shown that excessive activated autophagy moved toward autophagic cell death (Maiuri, Zalckvar, Kimchi, & Kroemer, 2007; Pei et al., 2016). Recently, increasing evidence suggested that activation of autophagy can enhance the sensitivity of anticancer therapeutics (Levy et al., 2017; Pei et al., 2016; Zhang, Yin, & Sui, 2018). Moreover, rapamycin, an autophagy inducer, has been approved by the U.S. Food and Drug Administration for cancer treatment (Gentzler, Altman, & Platanias, 2012). Therefore, the autophagic pathway might be a promising therapeutic target for cancer chemotherapy. BIX‐01294 (BIX; a diazepin‐quinazolin‐amine derivative) was discovered to be a selective inhibitor for euchromatic histone‐lysine *N*‐methyltransferase 2 (EHMT2) histone methyltransferase (HMTase) initially (Chang et al., 2009). Recently, BIX has been found to be an effective inducer of autophagy and even to induce cell death at a high dosage (Kim et al., 2013). Moreover, BIX has also been demonstrated to increase glioma cell radiosensitivity (Gursoy‐Yuzugullu et al., 2017). However, whether BIX can enhance the sensitivity of gastric cancers to chemotherapy and the potential mechanisms remain unknown.

Pyroptosis, characterized by cell swelling and pore formation, is a form of lytic cell death (Kovacs & Miao, 2017). The Gasdermin superfamily constitutes of conserved proteins, including gasdermin A, B, C, D, E, and DFNB59, most of which have now been shown to have pore‐forming activity and then induce pyroptosis (Ding et al., 2016). Gasdermin D (GSDMD) can be cleaved by activated caspase‐1 or caspase‐11/4/5 to release its N‐terminal pore‐forming domain (PFD; Kayagaki et al., 2015). The PFD oligomerizes to pore the membrane and then drive swelling and membrane rupture (Kayagaki et al., 2015; Liu et al., 2016). GSDME/DFNA5, a nonsyndromic hearing impairment gene, resembles the membrane pore formation effects of GSDMD in being cleaved by active caspase‐3 and induce characteristics of pyroptosis (Wang et al., 2017). Meanwhile, GSDME has been demonstrated to be highly expressed in gastric tumors and be cleaved by activated caspase‐3 in a previous study (Wang et al., 2018). However, the role of GSDME in chemotherapy for gastric cancers remains largely unclear.

In this study, we, first, confirmed that BIX could enhance the sensitivity of gastric cancer to chemotherapy. Moreover, we found that BIX combined Cis (BIX + Cis) could induce GSDME‐mediaed pyroptosis in gastric cancer. Finally, we demonstrated BIX‐induced autophagic flux play a vital role in enhancing the sensitivity of gastric cancer cells to chemotherapy.

## 2 METHODS

### 2.1 Cell culture and treatments

SGC‐7901 (gastric adenocarcinoma), AGS (gastric adenocarcinoma), and MKN‐45 (gastric carcinoma) cell lines were obtained from the National Infrastructure of Cell Line Resources (Chinese Academy of Medical Sciences, Beijing, China) and maintained in Roswell Park Memorial Institute (RPMI) 1640 medium (Corning) with 10% fetal bovine serum (Hyclone, Logan, UT), 100 U/ml penicillin and 100 mg/ml streptomycin (Gibco) and 2 mM l‐glutamine (Gibco). All cells were kept at 37°C in a 5% CO_2_ humidified incubator. SGC‐7901, MKN‐45, and AGS were pretreated with or without BIX (B9311; Sigma‐Aldrich) for 2 hr, and then treated with cis‐platinum (Cis; 1134357; Sigma‐Aldrich) at the indicated concentrations and different time points.

### 2.2 In vivo nude mouse model

The male BALB/c nude mice (6–8 weeks) were purchased from the Vital River Laboratory Animal Technology Co., Ltd. (Beijing, China). These animals were maintained in the Animal Facilities of Experimental Animal Centre of Jinzhou Medical University under pathogen‐free conditions. After being randomly divided into four groups (six mice per group), the mice were injected subcutaneously with wild type (WT) SGC‐7901 cells, *GSDME*‐KO SGC‐7901 cells or *Bcelin‐1*‐KO SGC‐7901 cells (2 × 10^6^ cells/10 µl phosphate‐buffered saline [PBS]) into each flank. When the tumor volume reached about 15 mm^3^, mice were intratumorally injected with either BIX (15 μg/mice/2 day) 2 hr before intravenous injection with Cis (1 mg/kg/2 day) for another 15 consecutive days. After the experiment, all mice were euthanized by the carbon dioxide method. Tumor volume was recorded as: *V* (mm^3^) = length (mm) × width^2^ (mm^2^)/2. All animal studies were approved by the Institutional Care and Use Committee, Experimental Animal Centre of Jinzhou Medical University (Jinzhou, China).

### 2.3 CRISPR‐Cas9 knockout (KO)

The CRISPR‐Cas9 technology to generate target gene KO cell lines was used, as previously described (Ran et al., 2013). In brief, the following single guide RNAs (sgRNAs) to *GSDME* or *Beclin‐1* were used. *GSDME*‐forward sg1, 5′‐CACCGTCGGACTTTGTGAAATACGA‐3′ and *GSDME*‐reverse sg1, 5′‐AAACTCGTATTTCACAAAGTCCGAC‐3′; *GSDME*‐forward sg2, 5′‐CACCGTCATCAGAGACTCTGCCGAG‐3′ and *GSDME*‐reverse sg2, 5′‐AAACCTCGGCAGAGTCTCTGATGAC‐3′; *Beclin‐1*‐forward sg1, 5′‐CACCG AGTGGCAGAAAATCTCGAGA‐3′ and *Beclin‐1*‐reverse sg1, 5′‐AAAC TCTCGAGATTTTCTGCCACTC‐3′; and *Beclin‐1*‐forward, sg2 5′‐CACCG GATCTTAGAGCAAATGAATG‐3′ and *Beclin‐1*‐reverse sg2, 5′‐AAAC CATTCATTTGCTCTAAGATCC‐3′. The pSpCas9 (BB)−2A‐Puro (PX459; 62988; Addgene) plasmid was digested and ligated with phosphorylated sgRNAs to construct the px459‐*GSDME*‐KO and px459‐*Beclin‐1*‐KO plasmid (Ran et al., 2013). Then, Lipofectamine® 3000 (Thermo Fisher Scientific) was used to transfect 2 × 10^5^ SGC‐7901 with 5 μg px459‐*GSDME*‐KO or px459‐*Beclin‐1*‐KO plasmid. After 48 hr, cells were cultured in RPMI‐1640 complete medium containing 2 μg/ml puromycin. After 72 hr, the clones were lifted and the expression of GSDME or Beclin‐1 was detected by western blot.

### 2.4 Lactate dehydrogenase (LDH) release assay

A CytoTox 96 Non‐Radioactive Cytotoxicity Assay Kit (Promega) was used to detect the release of LDH, according to the manufacturer's instructions. Briefly, the prepared substrate was equilibrated at room temperature and mixed with cell culture supernatant at a 1:1 ratio, then incubated at room temperature for 30 min. After incubation, Stop Solution was added and absorbance value at 490 nm was measured. To measure the total LDH lysis, 50 μl 9% (weight/volume) solution of Triton® X‐100 (CytoTox 96 Non‐Radioactive Cytotoxicity Assay kit, Promega) was added to the same number cells to measure the total LDH lysis.

### 2.5 Cell viability assay

SGC‐7901 (1 × 10^4^) or MKN‐45 (8 × 10^3^) were seeded into 96‐well plates and then treated with BIX or/and Cis at the indicated dosage and time points. After treatment, we washed cells with PBS, added 200 μl Cell Counting Kit‐8 mixture (Dojindo, Japan) into each well and incubated at 37°C for 1 hr according to the manufacturer's instructions. After incubation, 100 μl of the supernatant was used to measure the optical density values at 450 nm by an Infinite™ M200 Microplate reader (Tecan, Mannedorf, Switzerland).

### 2.6 Microscopy images

Cells treated with BIX or BIX combined Cis, 1 × 10^5^ SGC‐7901 cells were seeded into in 24‐well plates and treated as indicated. Static bright‐field images were photographed by using an Olympus IX71 microscope.

### 2.7 Flow cytometry analysis

After 12 hr treatment of Cis, SGC‐7901 cells were digested with trypsin digestion solutions without ethylenediaminetetraacetic acid (T1350; Solarbio), harvested and incubated with APC Annexin‐V (BD Biosciences) for 15 min at room temperature protected from light following the manufacturer's instructions. Washing cells with PBS was followed by propidium iodide (PI; 421301; Biolegend) incubation for 5 min. Two hundred microliter 1× binding buffer (BD Biosciences) was added to each sample and data were acquired by flow cytometer (BD Biosciences, CA).

### 2.8 Western blot

Treated cells were lysed in RIPA buffer (P0013C; Beyotime Company) containing a protease inhibitor (P8340; Sigma‐Aldrich) for 5 min and sonicated. After centrifuged, the supernatants were quantified using a BCA Protein Assay Kit (P0009; Beyotime) and 20 μg of the total protein extract were electrophoresed in sodium dodecyl sulfate and polyacrylamide gel and transferred onto a nitrocellulose filter membrane (Bio‐Rad). Membranes were blocked with QuickBlock™ Blocking Buffer (P0220; Beyotime) for 20 min and then incubated with primary antibodies including β‐actin (3700; Cell Signaling Technology), DFNA5/GSDME (ab215191; Abcam), cleaved caspase‐3 (9664; Cell Signaling Technology), LC3A/B (4108; Cell Signaling Technology), Beclin‐1 (3495; Cell Signaling Technology) or GSDMD (96458; Cell Signaling Technology) overnight at 4°C. After washing and incubating with the appropriate secondary antibodies (A0279 or A0288; Beyotime) for 1 hr at room temperature, the secondary antibodies were imaged by enhanced chemiluminescence (1:5,000; Thermo Fisher Scientific, MA) using a western blotting detection system (Bio‐Rad).

### 2.9 Statistical analysis

Statistical analysis and graphs were performed using GraphPad Prism 7.0 software (GraphPad Software, San Diego, CA). One‐way analysis of variance followed by Bonferroni's multiple comparisons post hoc test was used to compare the difference of three or more groups and Student's *t* test was used to compare the difference of two groups. All data was represented as the mean ± SEM of at least three independent experiments. In all statistical tests, a *p* < .05 was considered statistically significant.

## 3 RESULTS

### 3.1 BIX induces autophagic flux and autophagy‐associated cell dealth in gastric cancer cell lines

**Figure 1:**
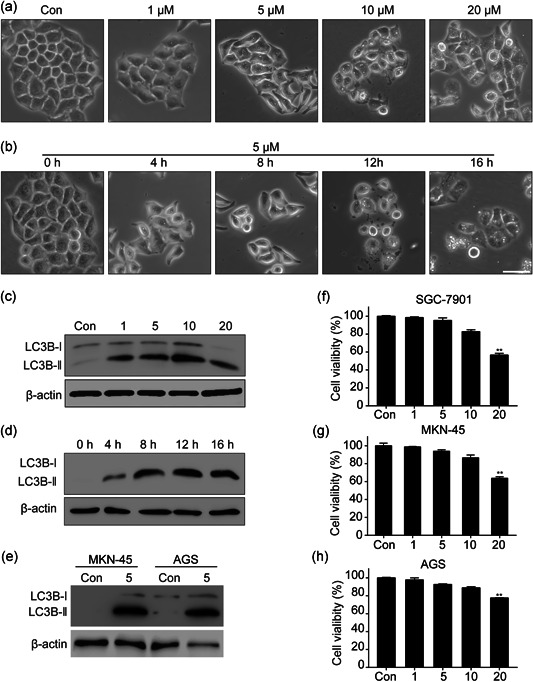
BIX induces autophagic flux in the SGC‐7901 cell line. (a) SGC‐7901 cells were treated with the indicated concentrations of BIX‐01294 (BIX) or vehicle (DMSO) for 12 hr, representative images are shown as photographed with a phase contrast microscope; scale bar = 50 μm. (b) SGC‐7901 cells were treated with 5 μM BIX or vehicle (DMSO) for different times. (c,d) Conversion of LC3B‐I to LC3B‐II was analyzed by immunoblotting. (e) Conversion of LC3B‐I to LC3B‐II in BIX treated MKN‐45 and AGS cells. (f) Cell viability of SGC‐7901 was measured by Cell Counting Kit‐8 mixture. (g) Cell viability of MKN‐45. (h) Cell viability of AGS. Data are presented as mean ± *SD* and at least three separate experiments in all studies. **p < .01 compared with control. DMSO, dimethyl sulfoxide

BIX has been reported as a strong chemical autophagy‐inducer in different tumor cell lines, however, whether it is able to induce autophagic flux in gastric cancer cells remains unknown. First, we used different doses of BIX to treat gastric cancer cell line, SGC‐7901, and found that BIX could induce the formation of large cytoplasmic vacuoles, which resembled autophagic vesicles in a dose and time point‐dependent manner (Figure 1a,b). The critical hallmark of autophagy, the conversion of the cytosolic protein form, LC3B‐I to the phagophore membrane‐bound LC3B‐II, could be also detected by immunoblotting in a dose‐ and time‐dependent manner, further supporting the fact that autophagic flux was activated by BIX treated SGC‐7901 cells (Figure 1c,d). The same phenomenon was also observed in other BIX treated gastric cancer cells lines, MKN‐45 and AGS cells (Figure 1e). In a previous study by Kim et al. (2013), they have demonstrated that a breast cancer cells line treated with 10 μM BIX for 24 hr could induce autophagy‐associated cell death, therefore we sought out to determine if similar results could be observed in BIX treated gastric cancer cells. Similarly, we found that in SGC‐7901, MKN‐45, and AGS cell lines, treated with 20 μM BIX for 24 hr, could decrease cell viability (Figure 1f–h). In conclusion, our data confirmed that BIX can strongly induce autophagic flux and autophagy‐associated cell death in gastric cancer cells.

### 3.2 BIX enhances the sensitivity of gastric cancer cell line to chemotherapy

**Figure 2:**
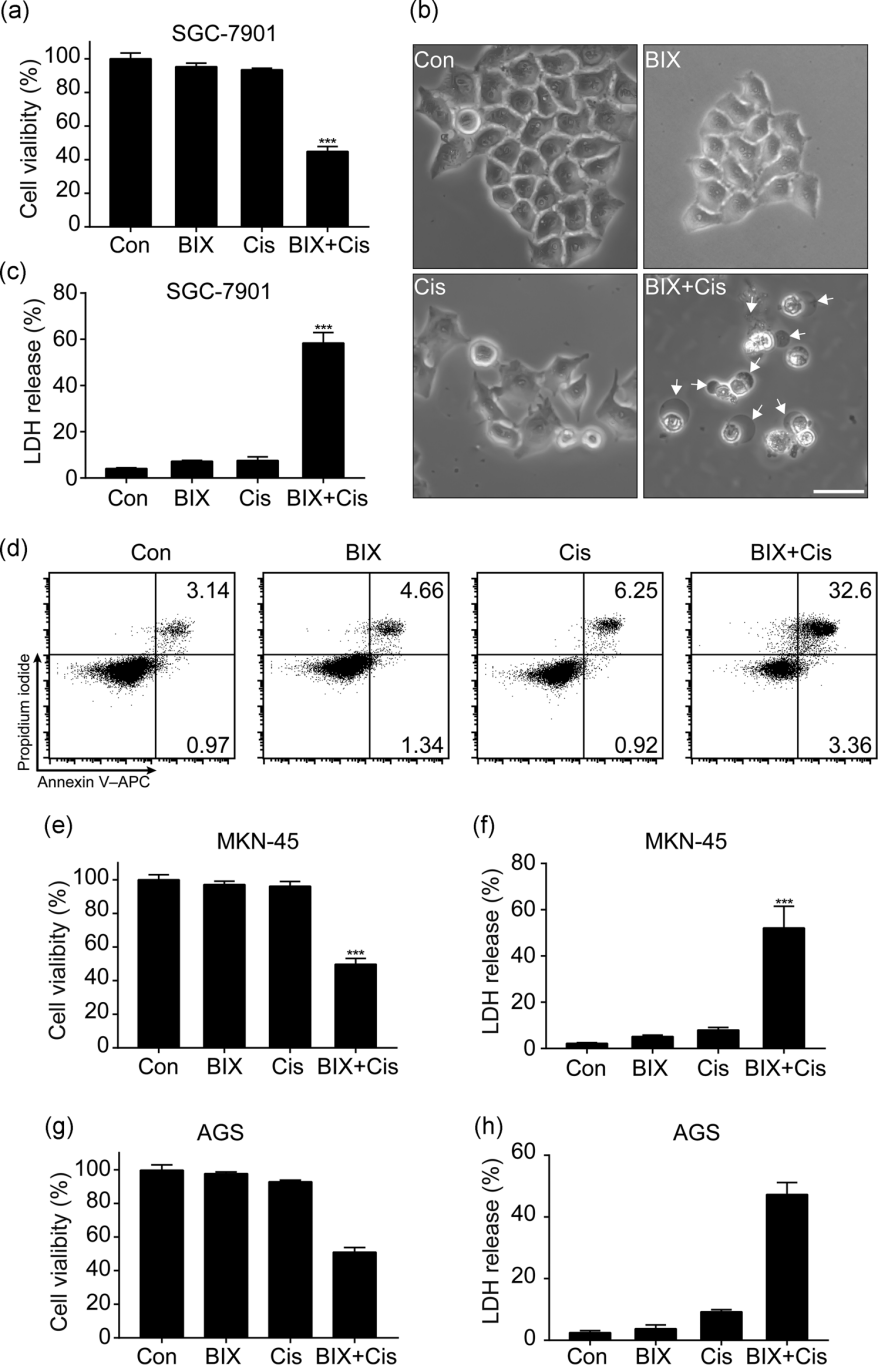
BIX enhances chemotherapy in SGC‐7901 cell line. SGC‐7901 cells were pretreated with 1 μM BIX for 2 hr then treated with 5 μM cis‐platinum (Cis) for 12 hr. (a) Cell viability. (b) Representative phase contrast imaging of cell death; scale bar = 50 μm. (c) Cytotoxicity of SGC‐7901 as measured by LDH release in the culture supernatants. (d) Flow cytometry of PI and Annexin V‐APC‐stained cells. (e,f) Cell viability and cytotoxicity of MKN‐45. (g,h) Cell viability and cytotoxicity of AGS. All data are representative of three independent experiments. Data are presented as mean ± *SD* and at least three separate experiments in all studies. ***p < .001 compared with control. LDH, lactate dehydrogenase; PI, propidium iodide

As mentioned above, BIX at high concentration could directly induce gastric cancer cell death, while at low concentration, it could only induce autophagic flux. We speculated that whether BIX at a relative low concentration could help to obtain gastric cancer cell sensitivity to chemotherapy. As we expected, SGC‐7901 cells pre‐treated with 1 μM BIX were particularly sensitive to the chemotherapy drugs Cis (5 μM) treatment with significantly decreased cell viability compared with only Cis treatment group (Figure 2a). To our surprise, the morphological changes of SGC‐7901 cells by BIX plus Cis treatment under a light microscope displayed cell swelling and large bubbles blew from the cell membrane identical to the image of pyroptosis, which is characterized by cell swelling and large pore formation on the plasma membrane (Aglietti & Dueber, 2017; Wang et al., 2017) (Figure 2b). To indicate that the cells undergo pyroptosis, we need to see some elements leak from the cytoplasm or enter into the cell, such as LDH and PI. Both the release of LDH and the entrance of PI into cells indicate that the integrity of cell membrane had been disturbed, which is the most important indicator of pyroptosis. Therefore, we measured the LDH release, which would leak into extracellular space from the cytosol after the loss of membrane integrity (Evavold et al., 2018). As expected, the LDH release was significantly increased in the BIX + Cis combined treated group but not by Cis or BIX alone (Figure 2c). Furthermore, the loss of membrane integrity also resulted in intracellular nucleic acids stained by the membrane impermeable dye PI (Evavold et al., 2018). Flow cytometry in Figure 2d showed that the percentage of Annexin V and PI double‐positive late stage apoptotic cells was higher in BIX + Cis combined treated group than the control and Cis or BIX treated groups. Similar results were obtained in other gastric cell lines, MKN‐45 and AGS, in which BIX + Cis combined treatment could also induce cell death and subsequent LDH release (Figure 2e–h). Collectively, these data suggest that BIX pretreatment would promote Cis induced gastric cancer cells pyroptosis.

### 3.3 BIX combined cis induced gastric cancer cells pyroptosis dependent on the cleavage of GSDME

**Figure 3:**
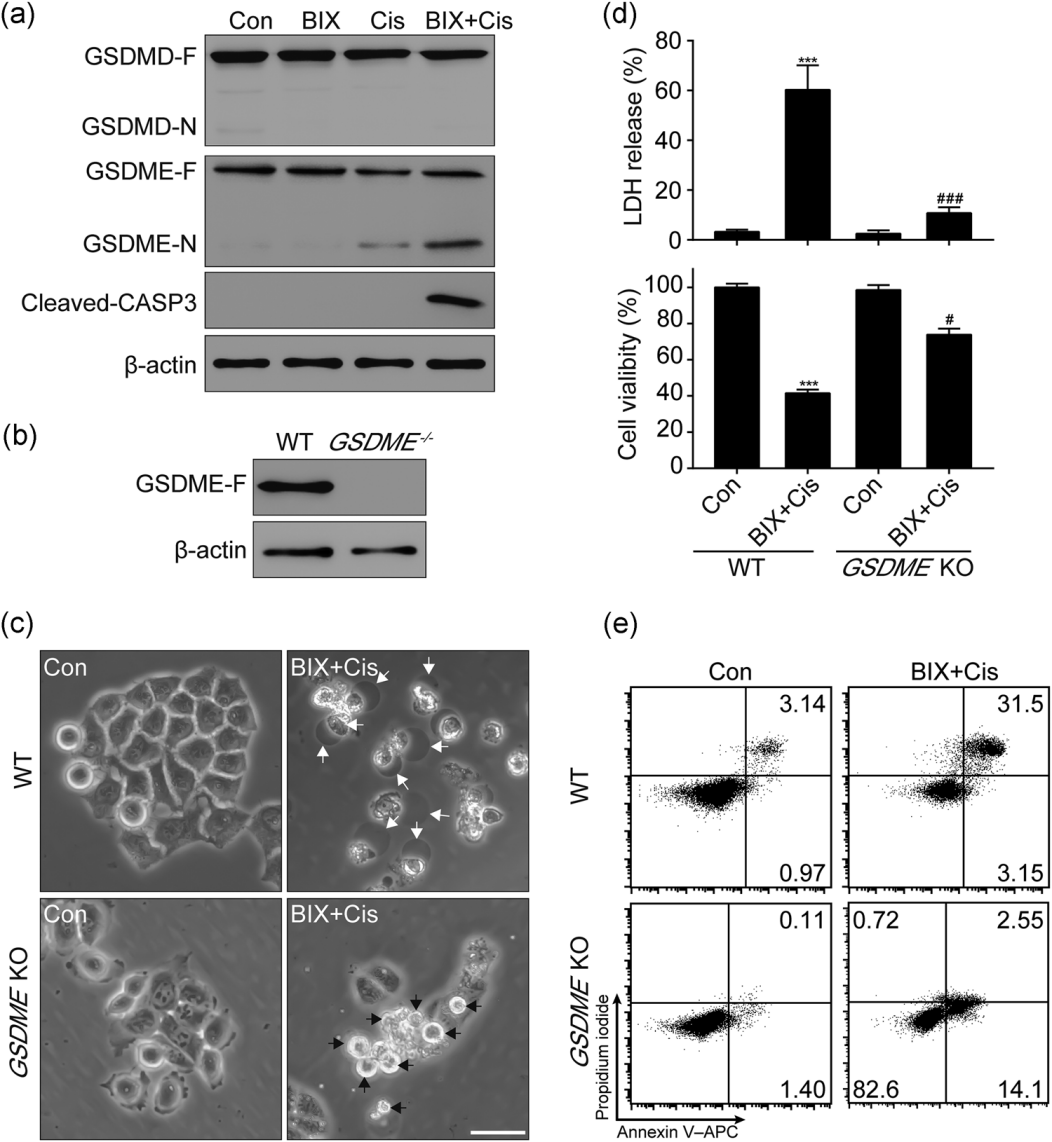
BIX combined chemotherapy drug to induce GSDME‐mediated pyroptosis in SGC‐7901. WT SGC‐7901 cells and GSDME‐KO SGC‐7901 (GSDME KO) cells were pretreated with 1 μM BIX for 2 hr then treated with 5 μM Cis for 12 hr. (a) GSDMD‐F, GSDMD‐N, GSDME‐F, GSDME‐N, and Cleaved CASP3 were detected by western blot in SGC‐7901. (b) Effects of GSDME knockout by using CRISPR‐Cas9 technology. (c) Representative phase contrast imaging of the different cell deaths in WT SGC‐7901 and GSDME‐KO SGC‐7901 cells; scale bar = 50 μm. (d) LDH release based cell death and cell viability. (e) Flow cytometry of Annexin V‐APC or PI and annexin V‐APC double stained cells. Data are presented as mean ± *SD* with at least three separate experiments in all studies. ***p < .001 compared with control, ^###^ *p* < .001 compared with WT BIX + Cis group. Cleaved CASP3, cleaved caspase‐3; GSDMD‐F, full‐length GSDMD; GSDMD‐N, GSDMD‐N terminal; GSDME, gasdermin E; GSDME‐F, full‐length GSDME; GSDME‐N, GSDME‐N terminal; LDH, lactate dehydrogenase; WT, wild type

GSDMD has been demonstrated to play a critical role in inducing pyroptosis by eliciting pore formation on the cell membrane (Tamura & Shiroishi, 2015). We first detected the full length and N‐terminal of GSDMD by immunoblotting to further explore the potential mechanism of BIX combined Cis induced gastric cancer cells pyroptosis. However, no cleavage of GSDMD (GSDMD‐N) after the treatment of BIX + Cis could be detected (Figure 3a). We then detected the cleavage of another member of the gasdermin family, gasdermin E (GSDME), which was reported to exert pyroptosis depending on the cleavage of caspase‐3 (Cleaved CASP3). Both N‐terminal of GSDME and Cleaved CASP3 were obviously detected by immunoblotting in the BIX‐Cis combined treated group (Figure 3a). Using the CRISPR‐Cas9 technology to KO the *GSDME* gene in SGC‐7901 cells (Figure 3b), we found that cell swelling and bubble blowing was diminished in the BIX‐Cis combined treated group, indicating that the cleavage of GSDME was necessary for BIX + Cis‐induced pyroptosis in SGC‐7901 cells (Figure 3c). Moreover, LDH release was completely blocked and cell viability was significantly increased in the BIX + Cis combined treated *GSDME* deficiency group compared with the WT group (Figure 3d). However, the *GSDME* deficiency could not completely block cell death after BIX + Cis treatment (Figure 3d). In the *GSDME*‐deficient group with BIX + Cis combined treatment, the percentage of Annexin V and PI double‐positive late apoptotic cells was significantly lower, but the percentage of Annexin V single‐positive early apoptotic cells was significantly higher than that in WT group (Figure 3e). These results indicated that BIX switched Cis‐induced gastric cancer cells apoptosis to pyroptosis depending on the cleavage of GSDME.

### 3.4 BIX enhanced anticancer effect by autophagic flux

**Figure 4:**
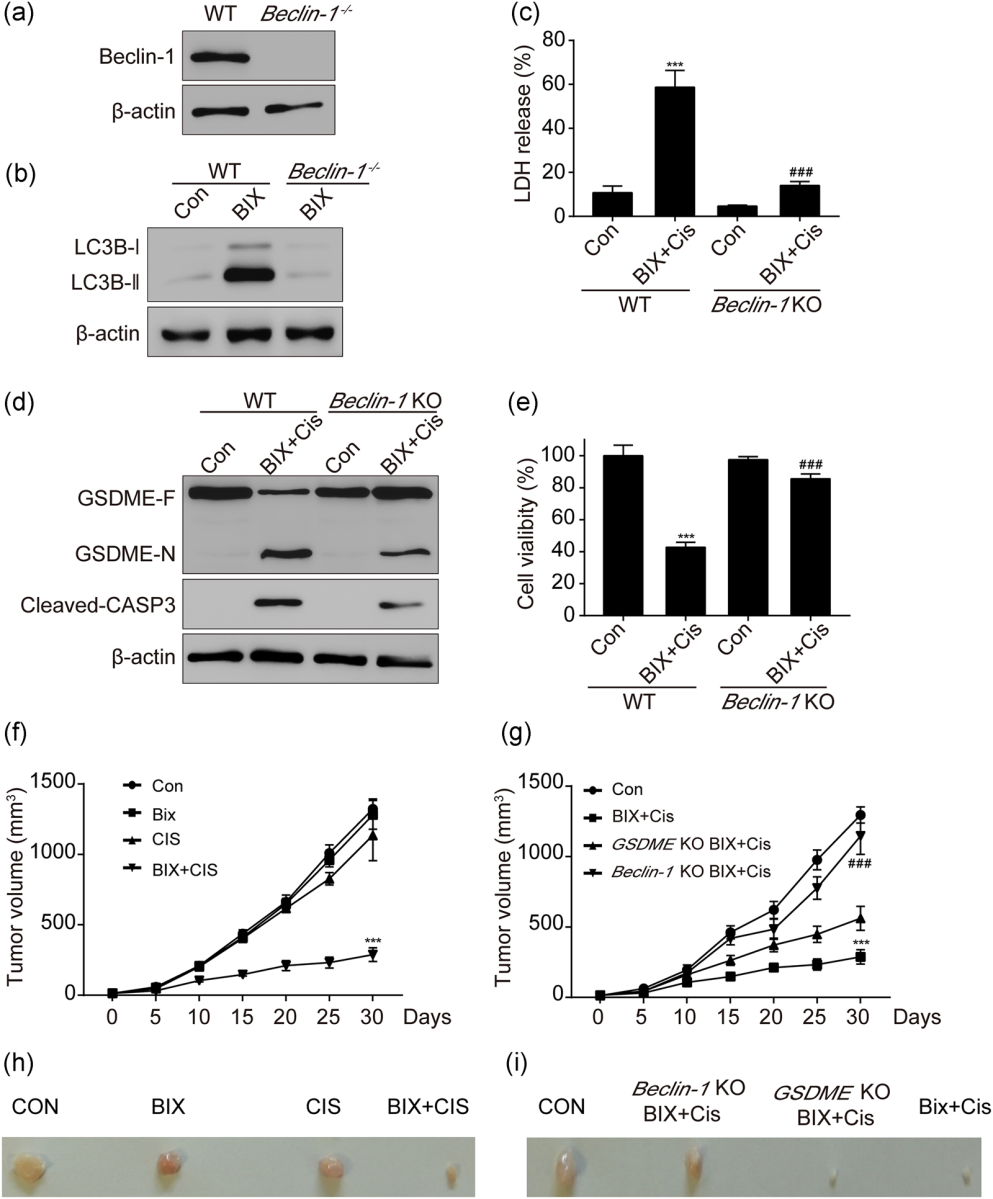
BIX enhanced anticancer effect by autophagic flux. WT SGC‐7901 cells and Beclin‐1 KO SGC‐7901 (Beclin‐1 KO) cells were pretreated with 1 μM BIX for 2 hr then treated with 5 μM Cis for 12 hr. (a) Effects of Beclin‐1 knockout by using CRISPR‐Cas9 technology in SGC‐7901 cell line. (b) Western blot analysis of conversion of LC3B‐I to LC3B‐II. (c) LDH release. (d) GSDME‐F, GSDME‐N, and Cleaved CASP3 were detected by western blot in WT SGC‐7901 cells and Beclin‐1 KO SGC‐7901 cells. (e) Cell viability. (e,g) Tumor volume after the treatment of BIX and Cis. (h,i) The representative image of tumor. Data are presented as mean ± SD and at least three separate experiments in all studies. ***p < .001 compared with control, ^###^ *p* < .001 compared with WT BIX + Cis group. GSDMD‐N, GSDMD‐N terminal; GSDME, gasdermin E; GSDME‐F, full‐length GSDME; LDH, lactate dehydrogenase

As demonstrated above, BIX can strongly induce autophagy related cell death, therefore, we proposed that BIX induced autophagic flux play a vital role on BIX + Cis triggered pyroptosis. To validate our hypothesis, we used the CRISPR‐Cas9 technology to knock out the *Beclin‐1* gene in SGC‐7901 cells, which regulates the initiation of autophagy and the results of immunoblotting confirmed that we knocked out the *Beclin‐1* gene successfully (Figure 4a). As expected, the conversion of LC3B‐I to LC3B‐II induced by BIX was obviously inhibited in the *Beclin‐1* KO group compared with the WT group (Figure 4b). Notably, BIX + Cis combined treatment induced LDH release and the cleavage of GSDME was significantly diminished in the *Beclin‐1* KO group (Figure 4c,d). Meanwhile, the cleavage of caspase‐3 was significantly blocked and cell viability was reversed by BIX + Cis combined treatment in the *Beclin‐1* KO group compared with that in the WT group (Figure 4d,e). These data suggested that BIX + Cis combined induced pyroptosi‐related cell death through the activation of autophgic flux in gastric cancer cells. Furthermore, we constructed an in vivo tumor model by injecting SGC‐7901 cells into nude mice and found that the tumor growth was significantly inhibited in the BIX + Cis treatment group compared with others (Figures 4f and 4h), however, the deficiency of *Beclin‐1* but not GSDME completely blocked the effect of BIX + Cis effects in vivo (Figures 4g and 4i).

## 4 DISCUSSION

BIX, a pluripotency inducer via EHMT2 inhibition, was used as a replacement of Oct3/4 in generating induced pluripotent stem cells from mouse fetal neural precursor cells in the absence of Sox2 (Shi et al., 2008). Recently, it is reported that BIX increases the transcription of *Beclin‐1* to activate autophagy and even induces autophagy‐associated cell death (Park et al., 2016). However, emerging evidence suggests that induction of autophagy can not only limit tumorigenesis in the long‐term, but also promote autophagic cell death and enhance the sensitivity of anticancer therapeutics in various types of cancer (Sun et al., 2017). The precise molecule mechanism of how BIX enhances anticancer therapeutics has not been elucidated yet. In Kim et al.'s (2013) study, they have found that BIX induced breast cancer autophagy‐associated cell death via EHMT2/G9a dysfunction and reactived oxygen species production (Kim et al., 2013). However, compared with 1 μM for inducing strong autophagic flux, 10 μM BIX may be too high to be applied in the clinic. In our study, we found that BIX can also induce autophagy of gastric cancer cells in a dose‐ and time‐dependent manner. Meanwhile, the ability of the BIX induced gastric cancer cell line, SGC‐7901, MKN‐45, and AGS death was increased with the dosage, which was similar to the effect of BIX on other cancers (Huang, Zou, Lin, Ma, & Huang, 2017; Zhu et al., 2018). Interestingly, we found that a low dose of BIX (1 μM) can help obtain the sensitivity of gastric cancer to a low dose of chemotherapy, which was further confirmed in vivo experiments. Overall, our results suggested that BIX might be a new promising targeted therapeutic agent in the clinic.

Pyroptosis, an inflammatory form of cell death, was initially found to protect organisms from invading pathogenic bacteria and microbial infections (Aglietti & Dueber, 2017). Recently, along with unceasingly thorough research, especially the finding of GSDME pore formatting during chemotherapy, pyroptosis has been proved to play a vital role in anticancer therapeutics (Rogers et al., 2017; Wang et al., 2017). Moreover, Wang et al.'s (2018) work and Lu et al.'s (2018) study have found that GSDME‐mediated pyroptosis may be a previously unrecognized mechanism to eradicate oncogene‐addicted neoplastic cells in gastric cancer and lung cancer. These prompted us to explore whether BIX combined Cis could also induce pyroptosis in gastric cancer. As expected, our results found that BIX + Cis combined treatment obviously induced pyroptosis in SGC‐7901, which was characterized by cell rupture and bubbles blowing from the membrane, and increased percentages of PI and APC annexin‐V double positive cells. Decreased LDH release and cell viability were not only observed in SGC‐7901 cells but also in MKN‐45 and AGS cells, further confirming the rupture of the membrane in BIX combined Cis treated gastric cancer cells. Furthermore, the cleavage of GSDME but not GSDMD was observed in the BIX + Cis combined treated group and the KO of *GSDME* significantly switched pyroptosis to apoptosis, which confirmed that BIX combined Cis induced pyroptosis in gastric cancer dependent on the cleavage of GSDME and caspase‐3. However, the deficiency of *Beclin‐1* in SGC‐7901 completely blocked BIX combined Cis induced pyroptosis as well as apoptosis, which suggested that BIX enhances the sensitivity of anticancer therapeutics in gastric cancer through the activation of autophagic flux. The in vivo results indicated that the KO of *GSDME or Beclin‐1* could both inhibit the effect of BIX + Cis treatment in vivo. However, the effect of *Beclin‐1* KO on BIX + Cis anticancer efficiency was stronger than that of *GSDME* KO, which was similar to what we found in vitro. These findings indicated that BIX‐Cis induced GSDME‐midiated pyroptosis through the activation of autophagic flux.

DFNA5 (GSDME) has been reported to induce high methylation in the putative gene promoter and is downregulated in many tumor cell lines, including breast, gastric, and colon cancer cell lines (Wang et al., 2017). But the human breast cancer line MCF‐7 and mouse mammary carcinoma cell line EMT6 have been found to express high levels of GSDME (Wang et al., 2017). And we found that SGC‐7901 also expresses a high level of GSDME, which is consistent with a recent study (Liu et al., 2020). Due to the heterogeneity of tumor cells, the high expression of GSDME may hint that GSDME probably exerts an alternative function in tumor cells. It has been reported that the higher expression of GSDME caused more extensive pyroptosis (Wang et al., 2017). Although Gsdme^−/−^ mice were often more healthy than wild‐type mice after the treatment of cisplatin (20 mg/kg), we did not observe obviously abnormality after the treatment of cisplatin at low concentrations (1 mg/kg/2 day). This may be because of the very high level of GSDME in SGC‐7901, which makes it more sensitized to BIX‐Cis treatment. BIX may change the state of GSDME methylation dependent inhibiting G9a, which sensitizes the tumor to chemotherapy. Therefore, the combination of BIX and Cis maybe a good treatment for the gastric cancer expressed very high level of GSDME or very low level of GSDME.

In conclusion, we were the first to report that BIX can induce autophagic flux and enhance the anticancer effect in gastric cancer. Moreover, our study also demonstrated that BIX combined Cis can induce GSDME‐mediated pyroptosis in gastric cancer cell lines. Furthermore, BIX combined Cis induced cell death dependent on the activation of autophagic flux.

## CONFLICT OF INTERESTS

The authors declare that there are no conflict of interests.

## AUTHOR CONTRIBUTIONS

G. W. conceived and designed the study. B. D., B. J., Y. L., and Y. L. performed the experiments. B. D. conducted data analysis and drafted the manuscript. G. W. revised and approved the manuscript. All authors have read and approved the final manuscript.

## Supporting information

Supplementary information
