# Supplementary material for: BIX‐01294 enhanced chemotherapy effect in gastric cancer by inducing GSDME‐mediated pyroptosis
Source: Cell Biol Int. 2020 Jun 8;44(9):1890–9. doi: 10.1002/cbin.11395 (PMC7496303; doi:10.1002/cbin.11395)
Supplement: Supplementary file 1 — Supplementary information [file CBIN-44-1890-s001.docx]

Dear Editors:

We are pleased to submit an original manuscript titled “BIX-01294 enhanced chemotherapy effect in gastric cancer by inducing GSDME-mediated pyroptosis” for consideration of publication as an “article” in Cell Biology International. This paper is new. Neither the entire paper nor any part of its content has been published or has been accepted elsewhere. All authors have approved to submit to Cell Biology International and no author has any conflict of interest.

Accumulating evidence suggests that targeting the induction of autophagy is regarded as a promising new strategy for cancer drug discovery. Our study firstly revealed that BIX-01294, an autophagy inducer, can sensibilize the gastric cancer to chemotherapeutics by induced GSDME-mediated pyroptosis through activating autophagic flux. We believe that this paper may be of particularly interest to the readers of your journal.

We hope that the editorial board of Cell Biology International would give our manuscript serious consideration.

Correspondence should be addressed to Gui-Jun Wang:

Department of General Surgery, The First Affiliated Hospital of Jinzhou Medical University, No.2, Section 5, Renmin Street, Guta District, Jinzhou City, Liaoning Province, PR China. Tel.: +86-0416-4197643; Fax: +86-0416-4197643. E-mail: lyfsyywgj@126.com

Looking forward to your decision,

With kind personal regards,

Yours Sincerely,

Gui-Jun Wang
